# Hydraulic characterization and start-up of a novel circulating flow bio-carriers

**DOI:** 10.1038/s41598-024-56857-x

**Published:** 2024-03-16

**Authors:** Xingyu Li, Guang Li, Yunyong Yu, Hongsheng Jia, Xiaoning Ma, Hong Yang, Prince Atta Opoku

**Affiliations:** 1 Key Laboratory of Songliao Aquatic Environment, Ministry of Education, Jilin Jianzhu University, Changchun, 130118 China; 2 Shanghai Investigation, Design & Research Institute Co., Ltd, Shanghai, China; 3 School of Environment, Harbin Institute of Technology, No. 92 West Dazhi Street, Nan Gang District, Harbin, People’s Republic of China

**Keywords:** Circulating flow carrier, Tracer test, Flow pattern, Hydraulic properties, Start-up, Efficiency, Biological techniques, Environmental sciences, Engineering

## Abstract

High-quality biofilm carriers are crucial for the formation of biofilm, but problems such as slow biofilm growth on the carrier surface have been troubling a large number of researchers. The addition of a carrier changes the flow state in the reactor, which in turn affects the microbial attachment and the quantity of microorganisms. Also, aerobic microorganisms need to use dissolved oxygen in the water to remove water pollutants. In this paper, a novel recirculating flow carrier with a hollow cylinder structure is proposed, with a certain number of hollow inverted circular plates placed at equal distances inside. In this paper, the hydraulic residence time, aeration volume, and the spacing of the inflow plates of the recirculating flow biofilm carrier, which are three important factors affecting the hydraulic characteristics of the reactor, are first investigated. At the same time, it was compared with the common combined carrier to find the optimal operating conditions for the hydraulic characteristics. Secondly, a reactor start-up study was carried out to confirm that the new recirculating flow biofilm carrier could accelerate the biofilm growth by changing the hydraulic characteristics. The results showed that under the same conditions, the hydraulic properties of the reactor were better with the addition of the recirculating flow carrier, with an effective volume ratio of 98% and a significant reduction in short flows and dead zones. The stabilized removal of COD, NH_3_-N, and TN in the reactor with the addition of the recirculating flow carrier reached about 94%, 99%, and 91% respectively, at the beginning of the 15th day, which effectively proved the feasibility of the recirculating flow carrier.

## Introduction

Generally speaking, the flow model of a reactor is divided into two categories: ideal flow model and non-ideal flow model. The ideal flow model is when fluid is in an ideal condition, where it flows from the two limit conditions: completely no back mixing of the pushover flow model and the maximum degree of back mixing of the full-mixed flow model^1^. However, in actual reactors, the deviation of the actual fluid flow from the ideal flow results in the formation of a non-ideal flow model due to the non-uniformity of the fluid flow rate in the reactor or non-ideal conditions such as short flow and dead zones. This is caused by internal configurations opposite to the main flow direction^2,3^. The study of the mechanism of the influence of hydraulic properties on wastewater treatment can provide an important basis for practical applications^4–6^. Complex hydraulic factors in bioreactors often affect the efficiency of microbial utilization of organic matter and effluent quality^7–9^. The hydrodynamic parameters can be determined by measuring the residence time distribution (RTD) of the reactor fluid^10^. At the same time, the RTD curve can be used to analyze the degree of deviation of the actual fluid from the ideal fluid in the reactor, which in turn guides the structural optimization and application of the reactor and carrier^11,12^.

**Figure 1:**
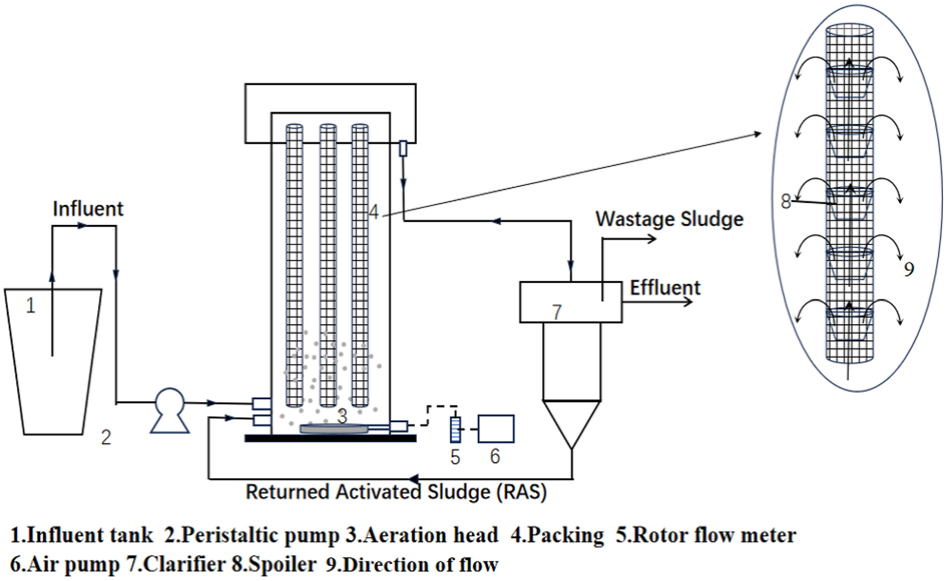
Experimental setup.

In recent years, with the development of biofilm water treatment technology, the existing biofilm carrier materials in terms of biocompatibility, stability, biofilm growth rate, treatment effect, and regeneration cannot meet the increasing emission standard requirements^13–15^. In the biofilm method of wastewater treatment, the formation of biofilm on the carrier surface is crucial, the problem of slow biofilm growth on the carrier surface has been troubling a large number of researchers^16–20^. The adhesion of microorganisms is the basis of biofilm formation. The structure of the carrier surface has a great influence on the adhesion, growth, and differentiation of microorganisms. The fluid flow pattern in the reactor also affects the degree of mixing between the biofilm carrier and the microorganisms. Observing its hydraulic characteristics, when the effective volume rate becomes higher and the short flow and dead zone are reduced, the better mixing degree of the carrier and microorganisms, and the contact is more adequate. The air bubbles generated in the reactor not only cause disturbances in the flow regime inside the reactor but also aerobic microorganisms need to utilize the oxygen dissolved in the water to remove pollutants from the water^21^. For the above reasons, this paper is innovative in the use of hollow deflector-shaped cylindrical structured circulating flow carriers. By hanging the biofilm carriers vertically in the cylindrical reaction device, the aeration head is set at the bottom of the reactor, the air is introduced into the carrier tube to form the role of airlift so that the sewage in the carrier tube forms a bottom-up flow pattern. The sewage in the carrier tube is discharged through the surface of the carrier tube at intervals (as shown in Fig. 1) so that the sewage forms a cycle in the inner and outer parts of the carrier tube to increase the sewage flow and the contact of the biofilm and improve the efficacy of sewage treatment. The purpose of this study is to investigate the hydraulic characteristics in the reactor under different operating conditions, hydraulic retention time (HRT), deflector plate spacing, and aeration through tracer experiments, and to find the best operating conditions based on the optimal hydraulic characteristics. As such, experiments were conducted to compare the differences in the filming performance of the two types of carriers (common combined carriers and circulating flow carriers) that changes in hydraulic properties brought about by recirculating flow carriers which accelerate the growth of carrier biofilms to obtain operating conditions that allow for faster biofilm growth and improved wastewater treatment.

## Materials and methods

### Hollow circulating flow carrier and reactor

The reactor used for the experiment was made of Plexiglas and its structure was cylindrical. The reactor was 125 cm high, had a bottom diameter of 30 cm, a total volume of 88.3 L, and an effective volume of 84.78 L. The reactor had a height of 125 cm and a bottom diameter of 30 cm. Inside the reactor is a suspended hollow circulating flow carrier with a height of 1 m. A deflector is placed at intervals inside the carrier, which is a hollow inverted circular table with a 60° beveled edge. The bottom of the reactor is installed with an aeration head, under the action of aeration, the sewage in the hollow circulation flow carrier forms a bottom-up flow pattern. The hollow circulation flow carrier part of the sewage in the guide plate under the guiding effect of the flow goes out of the carrier to achieve the cycle of the outflow flow pattern. The inlet flow is controlled by a peristaltic pump and the aeration is controlled by a rotameter. The carrier and reactor are cleaned at the end of each run. The start-up phase reactor with a settling tank for sludge return is shown in Fig. 1.

### 2 Analytical theory of RTD standardized curve

The residence time distribution curve (RTD curve) is a C(θ)-θ curve made by taking the standardized mass concentration C(θ) as the vertical axis and the standardized time θ as the horizontal axis, which is called the RTD curve^22–27^, and the area enclosed by this curve is one.

Standardized mass concentration formula:

1 $${\text{C}}\left(\uptheta \right)=\frac{{{\text{C}}}_{{\text{i}}}}{{{\text{C}}}_{0}},$$

where: C_0_ = total amount of tracer/effective volume of the reactor; Ci is the concentration of tracer in each outlet of the reactor at the time i, mg/L. The tracer concentration in each outlet of the reactor at the time i is the concentration of the tracer in each outlet of the reactor at the time i, mg/L.

Standardized time:

2 $$\uptheta =\frac{{\text{i}}}{{\text{HRT}}},$$

where HRT is the hydraulic residence time of the reactor, h.

Average length of stay:

3 $$\overline{t }=\frac{\sum_{0}^{\infty }{\text{tC}}\left({\text{t}}\right)\cdot \Delta {\text{t}}}{\sum_{0}^{\infty }{\text{C}}\left({\text{t}}\right)\cdot \Delta {\text{t}}},$$

Variance^28^:

4 $${\upsigma }_{t}^{2}=\frac{\sum_{0}^{\infty }{t}^{2}{\text{C}}\left({\text{t}}\right)}{\sum_{0}^{\infty }{\text{C}}\left({\text{t}}\right)}-{\left(\overline{t }\right)}^{2},$$

5 $${\upsigma }_{\theta }^{2}=\frac{{\upsigma }_{t}^{2}}{{(\overline{t })}^{2}},$$

where Eq. (5) is the uncaused variance. ${\upsigma }_{\theta }^{2}$ close to 1 is a fully mixed flow regime and close to 0 is an ideal push flow regime.

Effective floor area ratio (e)^29^:

6 $${\text{e}}=\frac{\overline{t}}{HRT },$$

The corresponding dead zone rate is

7 $$\frac{{V}_{d}}{V}=(1-{\text{e}})*100\mathrm{\%},$$

where ${V}_{d}$ is the dead zone of the reactor, L; V is the effective volume of the reactor, L. When e = 1, it is the ideal condition that there is no special fluid in the reactor; when e is between 0.9 and 1, there exists a small amount of dead zone or short flow in the reactor; when e is less than 0.9, the short flow phenomenon is serious or there exists a large dead zone in the reactor; when e is greater than 1, there exists vortex flow in the reactor.

Number of fully mixed units:

8 $$N=\frac{1}{{\upsigma }_{\theta }^{2}},$$

The larger N is, the more the number of series connections is, the more it tends to the ideal pushover flow state; on the contrary, the more it tends to the completely mixed flow state. That is, N = 1 is a completely mixed flow state, and N = ∞ is an ideal pushing flow state^30^. The resulting hydraulic efficiency index λ is^29^:

9 $$\uplambda ={\text{e}}*\left(1-\frac{1}{{\text{N}}}\right),$$

When λ is less than 0.5 it indicates poor hydraulic conditions, between 0.5 and 0.75 it indicates good hydraulic conditions, and greater than 0.75 it indicates very good hydraulic conditions.

Recovery of tracer^31^:

10 $$\upvarepsilon =\frac{\sum_{i=1}^{n-1}({C}_{i+1}+{C}_{i})({t}_{i+1}-{t}_{i})}{2*M}*Q,$$

where M is the total mass of the tracer added to the reactor and Q denotes the flow rate of the reactor fluid.

In this paper, Microsoft Excel 2010 was used for data processing, and Origin 2021 was used for the image processing.

### Tracer experiments

From the point of view of tracer selection criteria, the tracer must be easy to detect, easy to mix, not adhere to the surface of the equipment, not react with the host fluid, and have similar fluidity. Rhodamine B solution meets the above conditions and has the advantages of bright color, easy observation, and simple detection compared with tracers such as sodium chloride^32^. There are two methods of injection in the reactor, the pulse method and the stepper method, both of which are critical to the accuracy of the system. The pulse method involves the precise and instantaneous injection of a certain amount of tracer into the reactor through the fluid inlet. The curves generated by the pulse method are usually used to explain the mixing behavior of the system. In contrast, the stepper method usually involves the continuous addition of a tracer, usually after the fluid has stabilized. The disadvantage is that a large amount of tracer is required to effectively produce a significant and sustained change in concentration at the reactor outlet. Therefore, the pulse method was chosen to inject the tracer. The absorbance was measured using the UV spectrophotometer method and the tracer concentration was calculated from the standard curve (Appendix 1). The difference in the length of residence time of a fluid body is the residence time distribution (RTD) curve. The shape of the RTD curve allows for a visual determination of short flow and dead zones in the reactor. If the front end of the curve is steep it indicates the presence of short flow, while the trailing end of the curve is now evidence of a possible dead zone or stagnation zone in the reactor.

This study used a clear water experiment without activated sludge and the water used for the experiment was tap water. In each run, the inlet water flow was controlled by a peristaltic pump, and 500 mg/L of rhodamine solution was instantaneously injected into the reactor at 3% theoretical retention time after the reactor hydraulic state was stabilized. The entire sampling time should be 2.5–3 times the theoretical hydraulic retention time^33^, and samples should be taken at the reactor outlet starting at regular intervals. A minimum of 65 or more samples should be taken to fulfill the RTD curve plotting requirements. Firstly, based on the relevant literature, combined with the existing conditions in the laboratory, the three factors of hydraulic retention time (120 min, 180 min, 240 min), deflector plate spacing (10 cm, 15 cm, 20 cm, 35 cm, 50 cm), and aeration volume (0 L/min, 0.6 L/min) were selected to investigate the optimal working conditions of the circulating flow carrier’s reactor. Secondly, we compared and analyzed the effects on the hydraulic characteristics of the reactor under three different conditions: no carriers, adding common combined carriers, and adding circulating flow carriers.

### Start-up phase

Using the activated sludge from the secondary sedimentation tank of a sewage plant, under the conditions of influent COD of about 400 mg/L, ammonia nitrogen about 40 mg/L, HRT = 8 h, COD sludge load of 0.28–0.3 kg COD/(kg MLSS-d), the operation temperature of 25℃ and dissolved oxygen control of 3–4 mg/l, the differences in the performance of the membrane of the recirculating flow carrier (A) and the ordinary combined carrier (B) were compared. The test water was simulated domestic sewage, in which anhydrous sodium acetate, ammonium chloride, and potassium dihydrogen phosphate were used as the carbon, nitrogen and phosphorus sources in the feed water, and some trace elements (MnSO_4_-H_2_O 0.14 g/L, CuCl 0.19 g/L, ZnCl_2_ 0.05 g/L, H_3_BO_3_ 0.05 g/L) were also added to meet the normal metabolic demands of the cells. COD, ammonia nitrogen, and total nitrogen were determined by rapid digestion method, Nessler spectrophotometry, and potassium persulfate oxidation-ultraviolet spectrophotometry, respectively^34^.

## Results and discussion

### Factors affecting the hydraulic characteristics of the reactor

#### Hydraulic characteristics of the reactor at different HRTs

When the reactor volume is fixed, the change of HRT affects the change of flow rate in the reactor, the smaller the HRT, the larger the flow rate, and the higher the degree of turbulence of the fluid, which reduces the dead zone in the reactor and improves the volume utilization of the reactor. In this experiment, the effect of different HRT 120 min, 180 min, and 240 min) on the hydraulic characteristics of the reactor was investigated under the conditions of no aeration and 25 cm spacing of the deflector plate.

**Figure 2:**
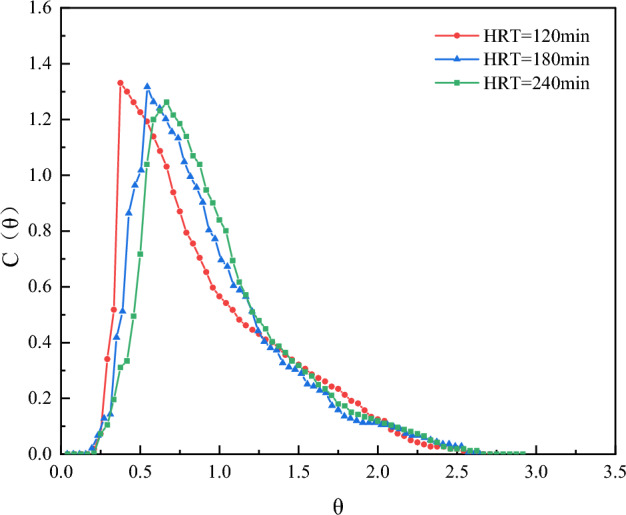
RTD curves for different HRTs.

As shown in Fig. 2, all three RTD curves have a single peak distribution. When the hydraulic retention time is small, the RTD curve peaks higher and steeper at the front of the curve, indicating the existence of a short flow phenomenon. The so-called short flow means that the water flow in the reactor does not follow the expected path of movement but chooses the shortest channel to flow out of the reactor quickly, which often occurs close to the inlet and outlet positions of the reactor. As the residence time increases, the peak of the RTD curve decreases gradually, making the peak-out time slightly shifted to the right, indicating that there is an increase in the hydraulic residence time which may increase the circulating flow inside and outside of the packed cylinder and improve the degree of short flow.

**Figure 3:**
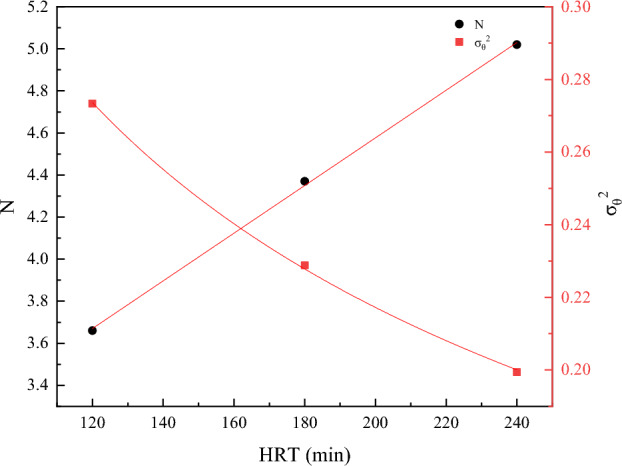
Fitted versus unfitted curves of N and σ^2^ concerning different HRTs.

From Table 1, the tracer recoveries were all above 100%, which was due to the different flow rates in the short stream and at the outlet of the reactor. With the increase of hydraulic residence time, the value of σ_θ_^2^ gradually decreases, and the value of N increases significantly, indicating that the flow pattern of the water in the reactor tends more and more to push the flow state. Plotting the two against HRT yields Fig. 3, a linear fit of N to HRT yields Eq. (11), and a nonlinear fit of σ_θ_^2^ values to HRT yields Eq. (12).

11 $${\text{y }} = \, 0.0{\text{1133x }} + { 2}.{\text{31, R2 }} = \, 0.{9993},$$

12 $${\text{y }} = { 2}.{381}0{\text{x}} - 0.{\text{4591, R2 }} = \, 0.{9994},$$

**Table 1:** Reactor hydraulic parameters for different HRTs.

Run	HRT (min)	σ_θ_^2^	N	e	λ	V_d_/v(%)	ε(%)
1	120	0.27	3.66	0.90	0.66	9.53	101.94
2	180	0.23	4.37	0.93	0.72	6.79	101.79
3	240	0.19	5.02	0.98	0.79	1.72	100.50

From the above equation, the N value is positively correlated with HRT, and increasing HRT can make the reactor fluid flow tend to push the flow state. The σ_θ_^2^ gradually tends to 0 with the increase of HRT, which further proves that the prolongation of HRT is favorable for the flow state in the reactor to be close to the pushing flow state. From the basic meanings of e and λ, as the HRT increases, the short flow decreases gradually and the hydraulic efficiency becomes better and better. Therefore, increasing the HRT reduces the short flow in the reactor, reduces the dead zone by increasing the effective reactor volume, and improves the hydraulic performance.

#### Analysis of the flow pattern inside and outside the carrier cylinder with different guide plate spacing

The spacing of the deflector plate affects the flow pattern inside and outside the carrier cylinder, and under the condition of hydraulic residence time of 240 min with no aeration, five spacings of 10 cm, 15 cm, 20 cm, 35 cm, and 50 cm were designed according to the length of the carrier.

**Figure 4:**
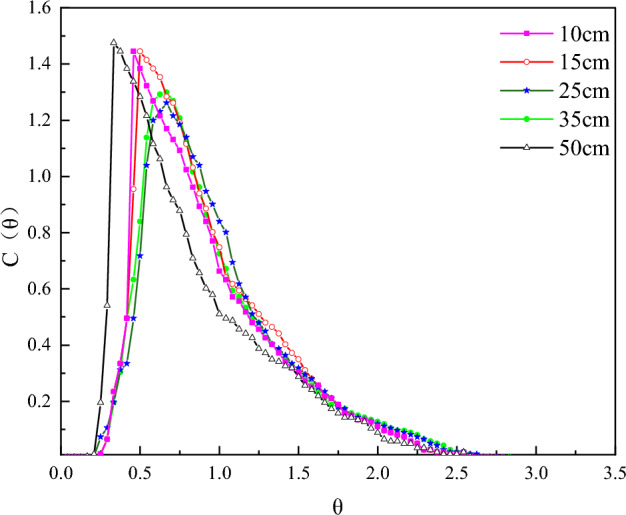
RTD curves for different deflector spacings.

From Fig. 4, the RTD curves for different spacings are all single-spike curves, and the RTD curves are shifted with the spacing of the deflector plates. The rightward shift of the RTD curve at 15 cm and 25 cm spacing indicates an improvement in the degree of short flow compared to the 10 cm spacing condition. When the spacing is further increased to 35 cm, the RTD curve is slightly shifted to the left, which indicates that increasing the spacing appropriately can promote the circulating flow inside and outside the carrier cylinder and reduce the short flow, but too large a spacing will exacerbate the degree of short flow in the reactor. By the same token, when the spacing is too large, the local liquids in the reactor do not form a circulating flow on the inside and outside of the carrier cylinder following the designed path. At a spacing of 50 cm, the curve continues to shift to the left and peaks at 0.3θ. The reason for this may be that the spacing is too large, and there is almost no circulating flow inside or outside the carrier cylinder, which produces a large number of short flows. In terms of peak height, the peak height is slightly reduced by decreasing the spacing, indicating that the spacing can be reduced to form a back-mixing phenomenon in the reactor.

From the reactor hydrodynamic parameters under different deflector spacings in Table 2, the tracer recoveries are above 99%, and the σ_θ_^2^ values and N values did not change much indicating that the guide plate spacings have a certain influence on the fluid flow pattern of the reactor, but the reactor as a whole still tends to pushing flow state. Compared with the parameter of 25 spacing, when the spacing was reduced to 10 cm, the e-value decreased by 5%, analyzing the reason may be due to the spacing being too small and the deflector plate also being too much which leads to the increase in circulating flow inside and outside of the carrier cylinder. It then increases the resistance of fluid along the water inlet direction, lifting the dead zone volume in the reactor. As spacing increases, the effective floor area ratio decreases by 14% when the spacing is increased to 50 cm. The same hydraulic efficiency index λ is the largest when the spacing of the deflector plate is 25 cm, which indicates that the proper spacing can increase the circulating flow inside and outside the carrier cylinder and reduce the dead zone to improve the hydraulic efficiency of the reactor, while the spacing is too small or too large will play the opposite role.

**Table 2:** Reactor hydrodynamic parameters at different deflector spacing.

Run	Aeration(cm)	σ_θ_^2^	N	e	λ	V_d_/v(%)	ε(%)
1	10	0.22	4.48	0.93	0.72	7.09	101.41
2	15	0.21	4.74	0.94	0.74	6.09	106.76
3	25	0.19	5.02	0.98	0.79	1.72	100.5
4	35	0.21	4.71	0.97	0.77	2.83	99.76
5	50	0.30	3.29	0.84	0.58	16.46	102.61

#### Flow analysis inside and outside the carrier cylinder under aeration conditions

Aerobic microorganisms need to utilize dissolved oxygen to remove pollutants from the water, thus requiring aeration equipment within the reactor^21^. This is to examine the effect of aeration on the fluid flow regime within the reactor due to the disturbance of the flow regime in the reactor caused by the bubbles produced^35,36^. The experiments were carried out under the conditions of HRT of 240 min and 25 cm spacing between the deflectors.

**Figure 5:**
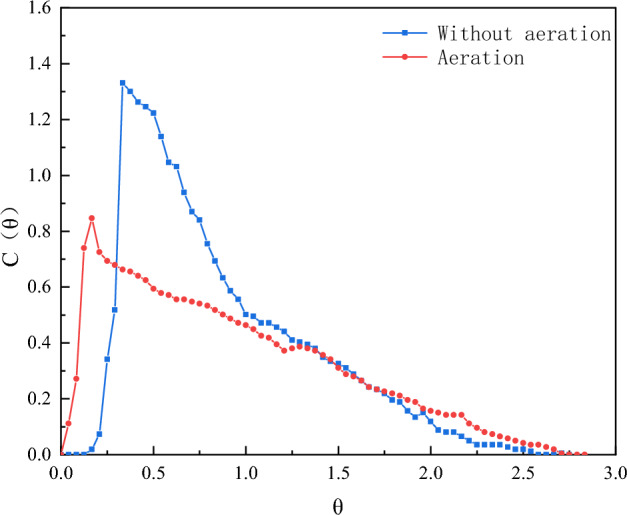
RTD curves with and without aeration.

From Fig. 5, the RTD curves with and without aeration are both single-spike curves, and both have steep front ends, indicating the presence of short flows. The drag at the back end of the curve was alleviated compared to the no-aeration condition. However, the curve as a whole shifted to the left, which may be due to the specificity of the reactor, the fluid direction in the reactor is the same as the aeration direction, and the liquid in the reactor is disturbed violently thus leading to the rapid mixing of the tracer. This creates the possibility that under the action of the deflector plate the carrier barrel will form a flat push flow, and the carrier barrel forms a mixed flow outside. In terms of peak height, increasing the amount of aeration resulted in a substantial decrease in peak height, indicating that aeration can create a back-mixing phenomenon in the reactor.

From the reactor hydrodynamic parameters under different aeration intensities in Table 3, the tracer recovery rate under the aeration condition is above 90% and not greater than 1, indicating that the aeration can make the tracer cluster diffuse more fully. Theoretical tandem complete mixing unit number N values are all greater than 1 and the value of σ_θ_^2^ is still closer to 0 demonstrating that the reactor has only a very small mixing situation. From the definition of λ, when the value of is between 0.5 and 0.75, it shows that the hydraulic state in the reactor is good, and the value of e is increased from 0.91 to 0.92. The reason for analyzing this is that the aeration causes the fluid in the reactor to move upward with the direction of the gas, thus making a part of the fluid overcome its gravity, and decreasing the volume of the dead zone in the reactor. In summary, it is shown that proper aeration can increase the liquid perturbation in the reactor, resulting in a fuller diffusion of the tracer and a reduction of the dead zone.

**Table 3:** Reactor hydraulic parameters with and without aeration.

Run	Aeration(L/min)	σ_θ_^2^	N	e	λ	V_d_/v(%)	ε(%)
1	0	0.19	5.02	0.98	0.79	1.72	100.5
2	0.6	0.30	3.32	0.90	0.63	10.18	95

#### Influence of carrier on the hydraulic flow regime

The placement of carrier material in the reactor facilitates the attachment of microorganisms and increases the number of microorganisms per unit volume^37^. Due to the special characteristics of the reactor, it is easy to make the tracer group mix too fast under the aeration condition, and it is not easy to conclude whether the circulating flow carrier has the advantage of hydrodynamic characteristics. Therefore, this experiment was carried out to compare and analyze the hydrodynamic characteristics of the reactor under the conditions of HRT of 240 min, 25 cm spacing between the guide plates of the recirculating flow carriers, and no aeration without adding carriers, adding common combined carriers and recirculating flow carriers.

**Figure 6:**
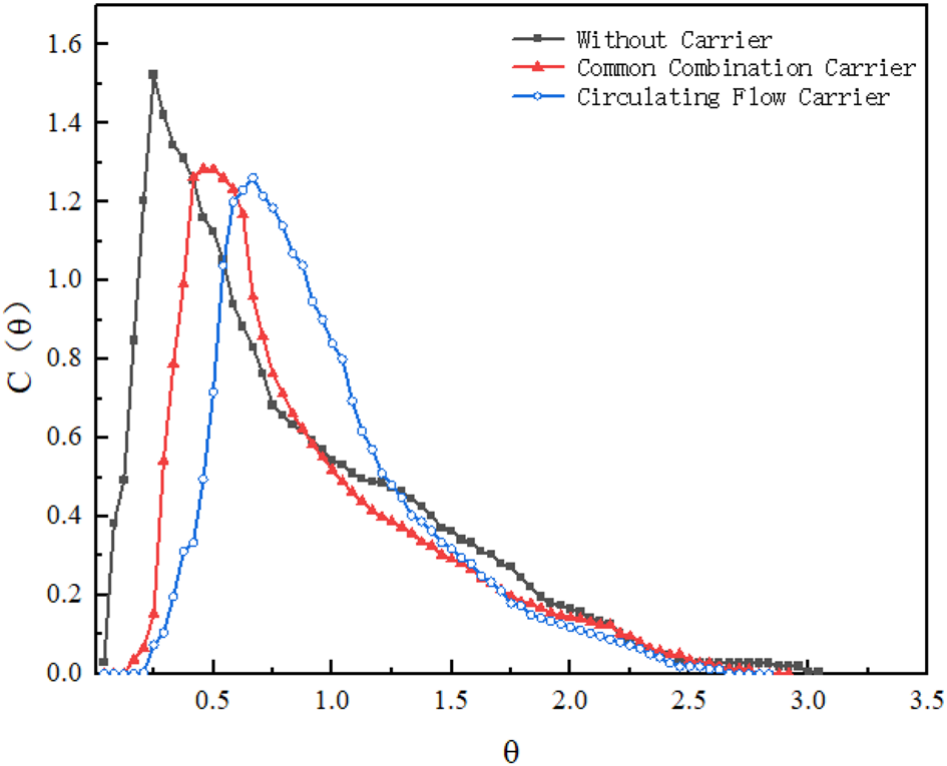
RTD curves for carrier.

Figure 6 represents the RTD profile of the reactor with and without carrier under the conditions of HRT of 240 min, aeration of 0 L/min, and 25 cm spacing of the deflector plates. In the absence of an added carrier, the tracer is propelled upward under the action of water flow, where part of the tracer rapidly flows out of the reactor in a short time resulting in short flow, while the remaining part is returned to mixing downward by the action of gravity and gradually flows out of the reactor with the prolongation of time, leading to the tracer test to be dragged out phenomenon^38^. Compared with that without carrier, the RTD curve shifted to the right side after adding carrier, and basically, no tracer was detected within 0.2θ, indicating that the short-flow phenomenon was significantly improved, and the tail-dragging situation was also alleviated^31^. Compared with the ordinary combined carrier, after adding the circulating flow carrier, the RTD curve distribution gets wider and wider, the peak height decreases, and the peak time continues to shift to the right, the reason for analyzing is that the addition of the circulating flow carrier changes the original water flow pattern in the reactor, and greatly weaken the short-flow phenomenon and the tail dragging situation.

As shown in Table 4, the tracer recoveries above 100% for all three effluent methods, which was caused by the different flow rates of the short stream and the effluent in the reactor, and the insufficient diffusion of the tracer mass. This resulted in the concentration of tracer at the effluent outlet being significantly higher than the average concentration of the reactor cross-section. The dead zone rate without carriers was as high as 14.62%, which decreased to 10.22% and 1.72% after carriers common combined carriers and circulating flow carriers respectively, indicating that there was a relatively large dead zone in the reactor without carriers and the fluid in the reactor tended to push the flow. After the addition of carrier σ_θ_^2^ which is closer to 0 and the N value increasing from 2.16 to 5.02, there is an indication that the fluid flow pattern in the reactor is more in favor of pushing flow state. The values of effective volumetric rate e and λ were increased by 1.15 and 1.71 times, respectively. This phenomenon indicates that the addition of a carrier weakens the short flow in the reactor, reduces the dead zones, and increases the effective volume of the reactor, which in turn improves the hydraulic performance. In summary, according to the RTD curves and the results of hydraulic parameters, circulating flow carriers can weaken the short flow in the reactor, reduce the dead zone to increase the effective volume of the reactor, and improve the hydraulic performance.

**Table 4:** Reactor hydraulic parameters for carrier.

Run	Carrier	σ_θ_^2^	N	e	λ	V_d_/v(%)	ε%
1	None	0.46	2.16	0.85	0.46	14.62	120.55
2	A	0.34	2.95	0.90	0.59	10.22	102.38
3	B	0.19	5.02	0.98	0.79	1.72	100.5

### Reactor start-up

Membrane initiation is one of the key factors affecting the operational performance of biological treatment systems. To compare the differences in the filming performance of the two types of carriers, a small test experiment was conducted to start up, the experiment was conducted for a total of 45 days. The growth rate of biofilm on the surface of the two types of carriers and the removal rate of different pollutants in the two sets of reactors were comparatively analyzed, to validate the high-efficiency performance of the recirculating flow carrier for wastewater treatment.

#### COD removal performance

**Figure 7:**
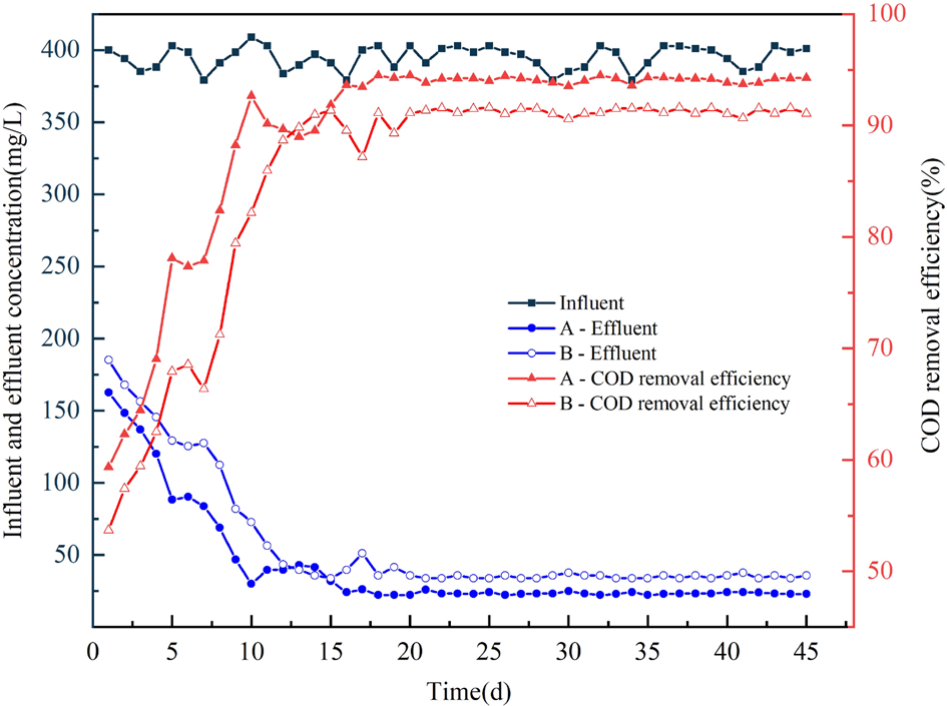
Effect of starting process on COD removal. Where A is the circulating flow carrier and B is the common combined carrier.

As can be seen from Fig. 7, the COD stabilization effluent of the two kinds of carrier was on the 16th and 20th day. The COD removal rate of the stabilized recirculating flow packing was significantly higher than that of the commonly combined packing (94% and 91% respectively. In the first 4 days of the reactor during the start-up period, the removal rate of the two types of carriers reached only 58–64%, and the effluent was greater than 100 mg/L. However, in the first four days of the reactor, the removal rate of the two types of carriers reached only 58–64%. However, the removal rate of the reactor with a circulating flow carrier increased slightly faster in the first four days, presumably because of its special guide plate structure, which makes it easy to form a circulating flow inside and outside the carrier, which is conducive to the utilization of organic matter by microorganisms, thus accelerating the growth of biofilm.

On the 12th day, the COD removal rate of both reactors reached 89%. However, the COD effluent of both reactors was not stable, indicating that the biofilm continued to grow and thicken at this time. There was no significant difference in COD removal between the two carriers during this period. With the continuous thickening of the biofilm, the effluent gradually stabilized. The COD removal rate was stable, indicating that the reactor start-up was successful.

#### NH3-N removal performance

**Figure 8:**
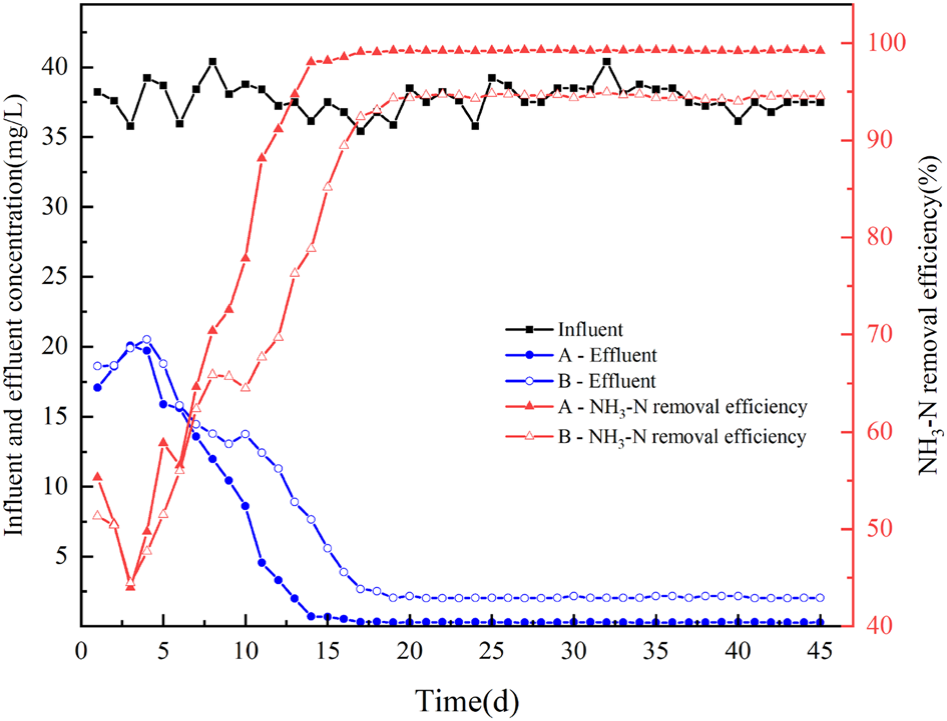
Effect of starting process on ammonia nitrogen removal. Where A is the circulating flow carrier and B is a common combined carrier.

It can be concluded from the graph analysis (Fig. 8), that in the first 3 days before the beginning of biofilm development, the nitrifying bacteria in the carrier colonization, and heterotrophic microorganisms compete fiercely, resulting in a slight decline in the ammonia nitrogen removal rate. At this time the two of the ammonia nitrogen removal rates were only about 50%, with the biofilm developed, for the ammonia nitrogen treatment rate to gradually increase. The ammonia nitrogen removal rate of group A increased from 50 to 73% by the 9th day, and gradually stabilized by the 13th day, while the ammonia nitrogen removal rate of group B increased from 44 to 66% and was gradually stabilized by the 16th day.

The above trend indicates that the reactor incorporating recirculating flow reaches the steady state faster than the reactor incorporating ordinary combined carrier during the start-up phase, indicating that the recirculating flow carrier can establish the nitrification system on the surface of the carrier faster, and the ammonia nitrogen removal rate of the reactor incorporating recirculating flow carrier is also significantly higher after the steady operation.

**Figure 9:**
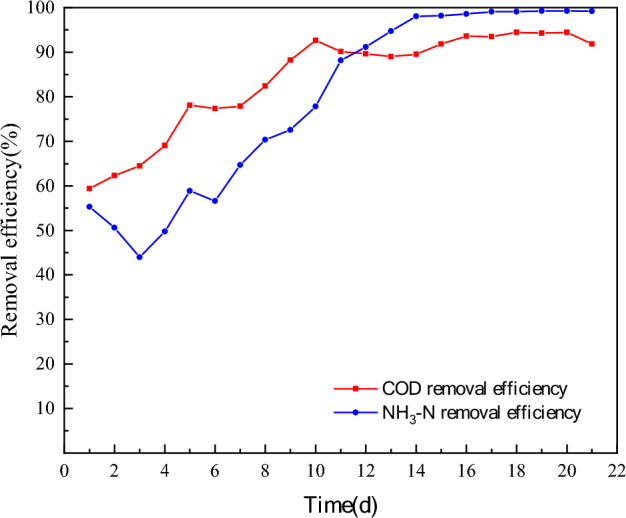
Variation of COD and ammonia nitrogen removal of the reactor with the addition of recirculating flow carriers during the start-up phase.

Re-analysis of Fig. 9 shows that the ammonia nitrogen removal rate of group A reached more than 80% of ammonia nitrogen in the 11th d, while the treatment rate of COD reached more than 80% of COD in the 8th d. Analyzing the reasons, this is mainly because the nitrite bacteria and nitrifying bacteria are autotrophic bacteria, that grow slowly have a long generation time, and need a long cultivation time to complete their proliferation and metabolism^39^. In the membrane stage, the carbon source of the influent water is more sufficient, which makes a large number of heterotrophic bacteria proliferate and grow, consuming a large amount of organic matter, at the same time, the number of heterotrophic bacteria has increased rapidly, occupying more ecological sites^40–42^, which affects the proliferation and growth of nitrifying bacteria and nitrifying bacteria. Only when the carbon source of the influent water was consumed by a large number of heterotrophic bacteria, and the carbon source in the system was low enough, then nitroso-chemical bacteria and nitrifying bacteria have a high proliferation rate^43^.

#### TN removal performance

**Figure 10:**
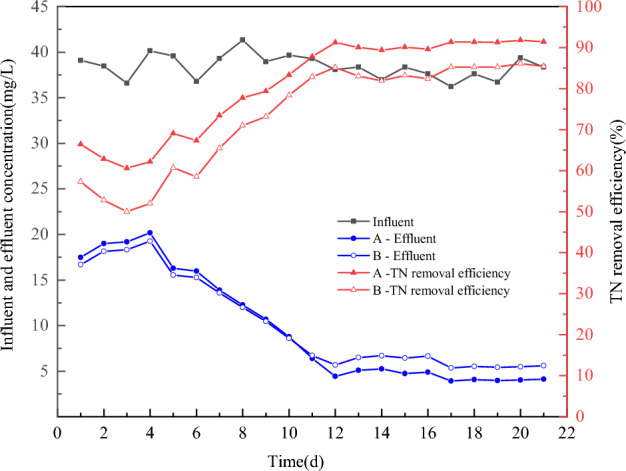
Removal effect of total nitrogen by the startup process. Where A is the circulating flow carrier and B is the common combined carrier.

As shown in Fig. 10, in the 1st d of the start-up period, the total nitrogen removal rates of both reactors were relatively low, 66.41% and 57.33%, respectively, and the effluent was about 17 mg/L, indicating that at this time, the nitrification system on the surface of the two types of carriers had not yet been established. Influence of total nitrogen removal in the reactor. With the growth of biofilm total nitrogen removal rate continues to rise, on the 10th day, both reactors’ removal rates reached more than 80%, but at this time are not stable. By the 12th day of operation, the total nitrogen removal rate of group A could already reach more than 90%. In contrast, Group B was run from day 17 until day 45 when the removal rate remained stable at about 85%. From the point of view of individual biofilm carrier, the circulating flow carrier may have faster and thicker biofilm maturation due to the internal circulating flow, and the biofilm is easy to form in aerobic, anoxic, and anaerobic microenvironments from the outside as well as the inside^44^.

## Conclusions

In this study, the residence time density distribution of the tracer in the reactor was investigated through flow tracer experiments, and the differences in the characteristics of recirculating flow carrier under different hydraulic residence times, different deflector plate spacings, and the presence or absence of aeration was also investigated to obtain the optimal operating parameters. The results clearly show that the hollow inflow-type cylinder structure circulating flow carrier is a viable option in terms of hydraulics.

1. Increasing the hydraulic residence time can make the flow pattern in the reactor close to the flat pushover flow, and the value of N is linearly related to the HRT, and the value of σ_θ_^2^ is negatively related to the HRT. Extending the hydraulic residence time can effectively increase the hydraulic efficiency of the reactor.
2. When the spacing of the deflector plate is 10 cm, part of the fluid may not reach the effect of circulation, which produces a negative effect. The phenomenon of short flow increases while the effective volume rate decreases. Correspondingly, the dead zone rate also increases, and when the spacing of the deflector plate is 25 cm, the effective volume rate which is close to 1, becomes a more suitable hydraulic parameter for this reactor. The σ_θ_^2^ values and N values did not change much indicating that the guide plate spacings have a certain influence on the fluid flow pattern of the reactor, but the reactor as a whole still tends to push the flow state.
3. Under the same parameter conditions, appropriate low aeration can increase the liquid perturbation in the reactor and reduce the number of tracer clusters to decrease the dead zone rate.
4. The fluid in the reactor tends to push the flow when there is no carrier and the short-flow phenomenon in the reactor is serious when there is no carrier, and there is a relatively large dead zone (14.62%); after adding two kinds of carrier respectively, the short-flow phenomenon can be weakened obviously, and the rate of dead zone can be reduced by 12.90%, 4.40%, and the effective volumetric rate of the reactor can be increased by 1.15 times and 1.05 times. The N values increased from 2.16 to 2.95 and 5.02, and the combined results showed that the fluid flow pattern in the reactor with the addition of recirculating flow packings was more in favor of advection flow.
5. In the membrane start-up stage, the circulating flow carrier was only used for 15 days for biofilm formation successfully, and the post-stabilization removal of COD, NH3-N, and TN reached 94%, 99%, and 91%, while the ordinary combination of carrier 20 days to stat-up successfully, the average removal rate of COD, NH3-N, and TN only 91%, and 94%, 85%, indicating that the circulating flow carrier biofilm formation faster, and the treatment effect is much better in the mud-film symbiosis system. It shows that the circulating flow carrier has a faster biofilm formation speed and better treatment effect in the mud-film symbiosis system. The results of the study proved that the hollow deflector-shaped cylindrical structured circulating flow carriers have good performance in terms of hydrodynamic performance and fast growth rate of surface biofilm.

The effect of circulating flow packing on microbial communities and on dissolved oxygen transfer efficiency was not investigated in this study. And the simulated domestic wastewater is controllable. Therefore, in future research work, we will further study the effect of the hollow inflow-type cylinder structure of the circulating flow carrier on the actual sewage treatment. The study of shock load resistance dissolved oxygen transfer efficiency, and the effect of packing on the microbial community in the reactor was carried out. Experiments were conducted using actual sewage and gradually expanded the scale of the reactor for the pilot study, in the pilot process for the actual working conditions to further optimize and evaluate the pollutant removal effect.

### Supplementary Information

Supplementary Information 1.

Supplementary Information 2.

## Data Availability

All data generated or analyzed during this study are included in this published article [and its supplementary information files].
