# Supplementary material for: Hydraulic characterization and start-up of a novel circulating flow bio-carriers
Source: Sci Rep. 2024 Mar 16;14:6384. doi: 10.1038/s41598-024-56857-x (PMC10944521; doi:10.1038/s41598-024-56857-x)
Supplement: Supplementary file 1 — Supplementary Information 1. [file 41598_2024_56857_MOESM1_ESM.docx]

Appendix


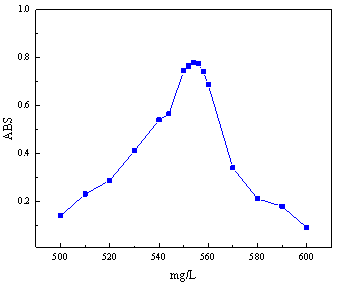

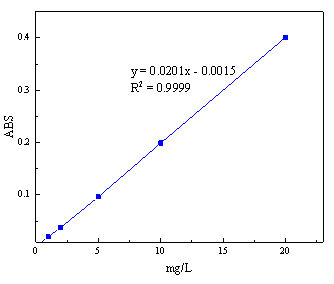


(a) (b)

Figure 1. (a) Absorption spectra of 1 mg/L rhodamine B; (b) Calibration curves for the determination of rhodamine B at its respective absorbance (λmax = 556 nm).
